# A global non-invasive methodology for the phenotyping of potato under water deficit conditions using imaging, physiological and molecular tools

**DOI:** 10.1186/s13007-021-00771-0

**Published:** 2021-07-23

**Authors:** M. Musse, G. Hajjar, N. Ali, B. Billiot, G. Joly, J. Pépin, S. Quellec, S. Challois, F. Mariette, M. Cambert, C. Fontaine, C. Ngo-Dinh, F. Jamois, A. Barbary, P. Leconte, C. Deleu, L. Leport

**Affiliations:** 1 UR OPAALE, INRAE, 17 Avenue de Cucillé, CS 64427, 35044 Rennes, France; 2 Centre Mondial de l’Innovation– Laboratoire Nutrition Végétale, Groupe Roullier, 18 Avenue Franklin Roosevelt, 35400 Saint-Malo, France; 3 Germicopa, 1 Allée Loeiz Herrieu, 29334 Quimper, France; 4 Bretagne Plant Innovation, Roudouhir, 29460 Hanvec, France; 5 UMR IGEPP, INRAE, Institut Agro-Agrocampus Ouest, Université de Rennes 1, Domaine de la Motte, 35653 Le Rheu, France

## Abstract

**Background:**

Drought is a major consequence of global heating that has negative impacts on agriculture. Potato is a drought-sensitive crop; tuber growth and dry matter content may both be impacted. Moreover, water deficit can induce physiological disorders such as glassy tubers and internal rust spots. The response of potato plants to drought is complex and can be affected by cultivar type, climatic and soil conditions, and the point at which water stress occurs during growth. The characterization of adaptive responses in plants presents a major phenotyping challenge. There is therefore a demand for the development of non-invasive analytical techniques to improve phenotyping.

**Results:**

This project aimed to take advantage of innovative approaches in MRI, phenotyping and molecular biology to evaluate the effects of water stress on potato plants during growth. Plants were cultivated in pots under different water conditions. A control group of plants were cultivated under optimal water uptake conditions. Other groups were cultivated under mild and severe water deficiency conditions (40 and 20% of field capacity, respectively) applied at different tuber growth phases (initiation, filling). Water stress was evaluated by monitoring soil water potential. Two fully-equipped imaging cabinets were set up to characterize plant morphology using high definition color cameras (top and side views) and to measure plant stress using RGB cameras. The response of potato plants to water stress depended on the intensity and duration of the stress. Three-dimensional morphological images of the underground organs of potato plants in pots were recorded using a 1.5 T MRI scanner. A significant difference in growth kinetics was observed at the early growth stages between the control and stressed plants. Quantitative PCR analysis was carried out at molecular level on the expression patterns of selected drought-responsive genes. Variations in stress levels were seen to modulate ABA and drought-responsive ABA-dependent and ABA-independent genes.

**Conclusions:**

This methodology, when applied to the phenotyping of potato under water deficit conditions, provides a quantitative analysis of leaves and tubers properties at microstructural and molecular levels. The approaches thus developed could therefore be effective in the multi-scale characterization of plant response to water stress, from organ development to gene expression.

**Supplementary Information:**

The online version contains supplementary material available at 10.1186/s13007-021-00771-0.

## Introduction

Potato (*Solanum tuberosum* L.) is cultivated on every continent other than Antarctica. With a yield of 374 million Mt (Metric ton) in 2017, it is the world’s fourth most produced crop after sugar cane (1851 million Mt), maize (1164 million Mt) and wheat (773 million Mt) (FAOSTAT2020. Available from: fao.org/faostat). Its global production has grown steadily for the last three decades (by around 40%). As an important staple food, it is grown in over 100 countries around the world for its adaptability to a wide range of environments. However, as for many crops, potato cultivation is under threat from global heating. Water stress is the main cause of yield loss in field crops and climate forecasts suggest that this problem will worsen in the coming years. Compared to other field crop species, potato is considered to be relatively sensitive to water stress [1–3]. One reason for this sensitivity is its shallow and sparse root system [4, 5]. Water stress causes a decrease in the number [6] and size of tubers [7] and a decrease in their quality due to biotic and abiotic disorders (scab, growth crack, hollow heart, etc.). The timing, duration and severity of the water deficit have a significant effect on stress expression. Additionally, a proportion of the expression is linked to tuber formation and growth which occurs underground. This explains why, although the consequences of water stress on potato yield are well known, the effects of dehydration at key stages of sensitivity on yield are poorly understood. The development of effective phenotyping tools, from microscopic to macroscopic level, especially for underground tubers, is a key bottleneck in deciphering and acquiring a better understanding of the effects of water stress on potato growth and its impact on yield.

Plants respond to drought by initiating multiple physiological and metabolic adjustments that form a complex network of cellular and molecular processes. This includes the dynamic shifting of regulatory responses during transcription and protein expression, consequently affecting numerous biochemical pathways and eventually leading to phenotypic changes [8–10]. In the last decade, high-throughput phenotyping tools have seen substantial development so that precise monitoring of the progression of morphological parameters under water deficit [11, 12] can be undertaken. Equally, other tools have now become available for the in situ characterization of leaf and tuber development and its disturbance under stress. Indeed, the capacity of Nuclear Magnetic Resonance (NMR) relaxometry to evaluate in detail the water distribution associated with the cell and tissue structures of oilseed rape, tobacco and lettuce leaves has been recently demonstrated [13–15]. NMR transverse relaxation time, which is particularly sensitive to variations in the water properties of plant tissues, was used to study changes in cell water status and distribution. As was demonstrated for different plant tissues, including leaves [13, 16], multi-exponential transverse relaxation times reflect the differences in the physical and chemical properties of water in different cell compartments under conditions where diffusion exchange of water molecules between compartments is relatively slow. It has been shown that in hydrated plant tissues (fleshy fruits, tubers, leaves) the longest T_2_ peak with the highest relative intensity is generally associated with vacuolar water [16], which is the largest pool of cell water. In the particular case of leaves, in the course of senescence, the single vacuolar peak splits into two peaks as a result of differential leaf hydration and cell enlargement between the palisade and spongy layers. Following the split, the T_2_ value of palisade cells (which have the longer T_2_ value) increases while that of spongy cells remains stable. Further, throughout plant development during the vegetative stage, the split is seen to progress from the bottom to the top of the canopy [17]. This pattern of T_2_ evolution reflects the orderly and progressive process of sequential leaf senescence. In the case of N depletion, it has been shown that lower tolerance to this stress was associated with a higher impact on senescence-associated structural modification patterns.

In Magnetic Resonance Imaging (MRI), the image contrast can be adjusted to provide anatomical information for an intact plant organ or to map the spatial distribution of relaxation parameters. One application is root imaging as an additional value in plant phenotyping [18]. MRI has been used to monitor the development of three-dimensional (3D) root architecture [18], to identify active roots for water uptake [19], and to evaluate pathogen-induced root damage [20]. In addition, 3D MRI images have allowed comparison of the internal physiology of chilled and non-chilled tulip bulbs during storage and after planting [21, 22]. This revealed that normal water balancing between different subcellular compartments (vacuole and cytoplasm) associated with water membrane permeability was a key process for the healthy development of tulip bulbs. A similar study found it possible to identify markers of dormancy release, a process that is not physiologically visible, by means of MRI images of tulip bulbs [21]. To the best of our knowledge, the growth kinetics of potato tubers are presented for the first time in this study.

In order to understand observed morphological changes, it is possible to combine phenotyping with physiological and genotyping analysis. For instance, plant response to water stress can be monitored by assessing leaf water relations and by measuring leaf-water potential, osmotic potential and relative water content, while yield components can be monitored by quantifying total biomass, potato tuber numbers and tuber fresh weight. Plants perceive water deficit signals through multiple signal transduction pathways which subsequently activate different drought-responsive genes producing adaptative resistance to stress [23]. A crucial role in the transduction of these stress signals is played by phytohormones, specifically abscisic acid (ABA) [24]. Stress signal perception and the consequent expression of drought-responsive genes involve different transcription factors which could be either ABA-dependent or ABA-independent [25]. Here, metabolomics and molecular tools provide the means to generate datasets for the study of changes in gene expression and metabolites resulting from drought stress.

In this study, a holistic approach was adopted towards potato plant responses to drought, focusing on the foliar parts of the plant as well as the tubers and combining both destructive and non-destructive methods in order to investigate the effects of water stress on the whole potato plant throughout the period of its lifecycle development between planting and harvest. This approach brought together breeders, geneticists, physiologists, physicists and agronomists to pool their expertise and to unlock information on potato plant response to the multifactorial effects of water stress. The plants’ physical environment was characterized by measuring air temperature and installing humidity and light sensors, while the measurement of the water deficit was finely calibrated by weighing the pots in order to quantify water use and tensiometers to monitor soil dehydration. NMR was used to evaluate the impact of water stress on the structural modification of leaves during development and MRI was used for underground monitoring of the number and volume of tubers. High throughput phenotyping tools were used to quantify changes in shoot morphology and color, using top and side view cameras. Abscisic acid (ABA) was quantified and the effect of water stress on gene expression was assessed for ABA and other drought-responsive genes. Gene expression analysis was performed, selecting ABA-dependent AREB (ABA-responsive element binding protein) transcription factors (*StAREB1* and *StAREB2*) and ABA-independent DREB (drought-responsive element binding) transcription factors (*StDREB1* and *StDREB2*) that bind to drought-responsive cis-acting elements and are induced by dehydration. Moreover, specific drought-inducible genes coding for Late-Embryogenesis Abundant (LEA) proteins (for example dehydrins) and chaperone proteins (heat shock proteins) such as *StDHN1*, *StTAS14*, *StERD7*, *StRD22* and *StHSP100* involved in adaptive stress responses were also analyzed by real-time PCR-based gene expression analysis. Using this integrative approach, the present study aims to establish a methodology for investigating the effects of drought on potato development, employing modern tools and innovative methods in combination with more traditional water stress evaluation techniques. Indeed, the effects of water stress can be characterized in an integrated manner at different plant levels from the whole plant (plant growth, water use, photosynthesis area) to yield components (tuber number and size, …). Additionally to these data, this holistic approach allows to provide the insight on plant functioning under water stress, from organ and cell physiology to gene expression. Therefore, deeper analysis will be possible for improving the understanding of the plant’s response to drought.

## Results

### High-throughput phenotyping parameters

**Fig. 1:**
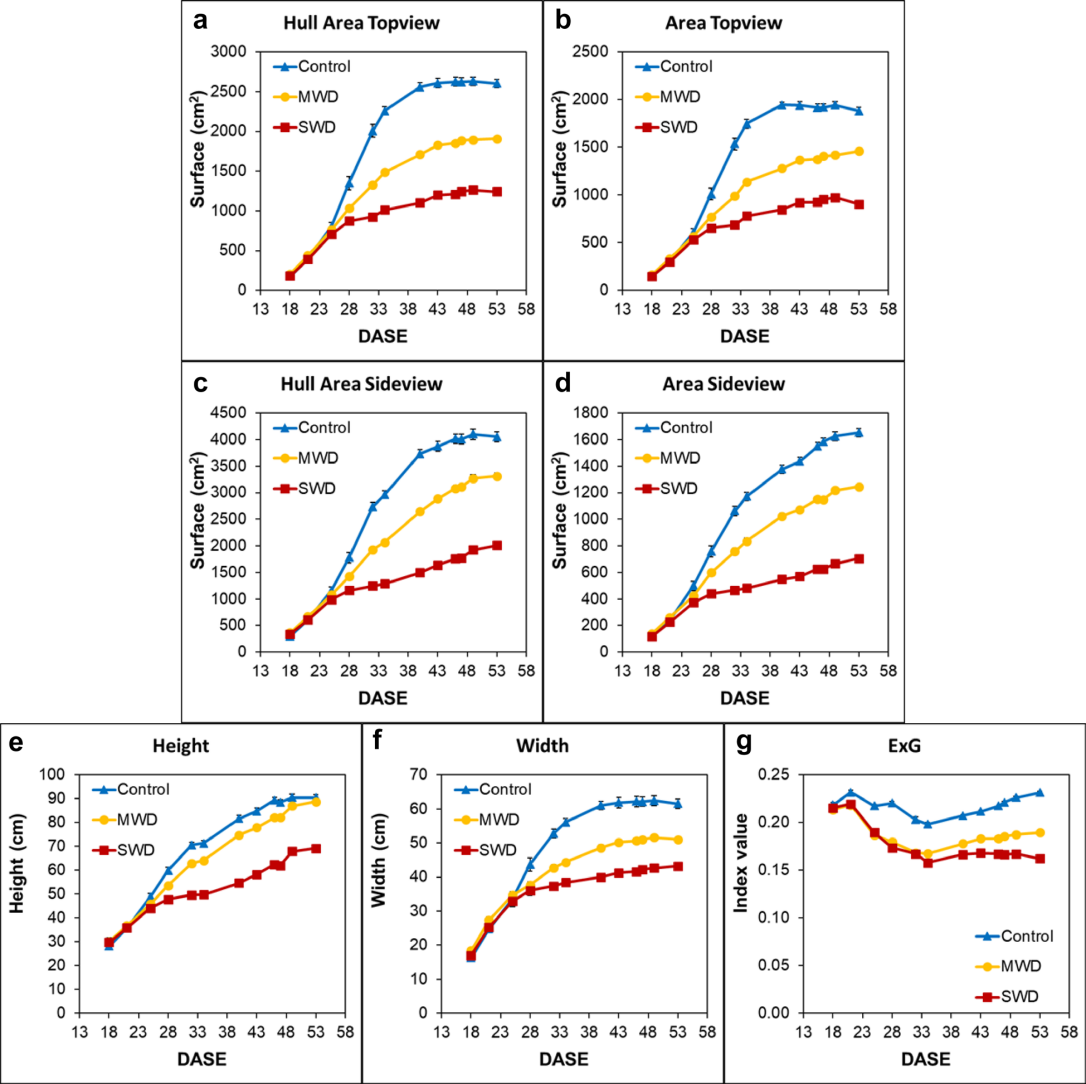
Impact of water stress on phenotyping parameters. Evolution measured in Days After Shoot Emergence (DASE) of **a**, **b** the hull area and projected area from the top view camera (leaf coverage), **c**, **d** the hull area and projected area from the side view camera, **e**, **f** height and width from the side view camera and **g** the ExG index. All parameters were computed from the day of transplantation into the soil to the day of harvest. For all of the mentioned parameters, a significant difference between the three conditions was observed from 34 to 53 DASE. Additional file 1: Table S1 for further details regarding statistical data

Three water regimes were tested first, (i) an optimal water supply (Control) condition corresponding to soil watering at 70% of field capacity, (ii) a Mild Water Deficit (MWD) condition corresponding to 40% of field capacity and (iii) a Severe Water Deficit (SWD) corresponding to 20% of field capacity. The impacts of water deficit throughout the potato plants’ growth period were monitored by evaluating several morphological and color parameters (Fig. 1). It was observed that all architectural parameters respond to the water gradient especially the biomass development represented by hull areas (from the top view or side view) from the 25th day after shoot emergence (25 DASE) (Fig. 1a–d). However, the height was only impacted by SWD (Fig. 1e). On the Excess Green index (ExG), it was noticed that the value decreased with the water deficit level (Fig. 1g). This can be explained by the decrease in chlorophyll pigments, as the green reflectance was lower than the standard chlorophyll activity for Control conditions.

### Variation of stress levels modulates ABA and drought-responsive genes

**Fig. 2:**
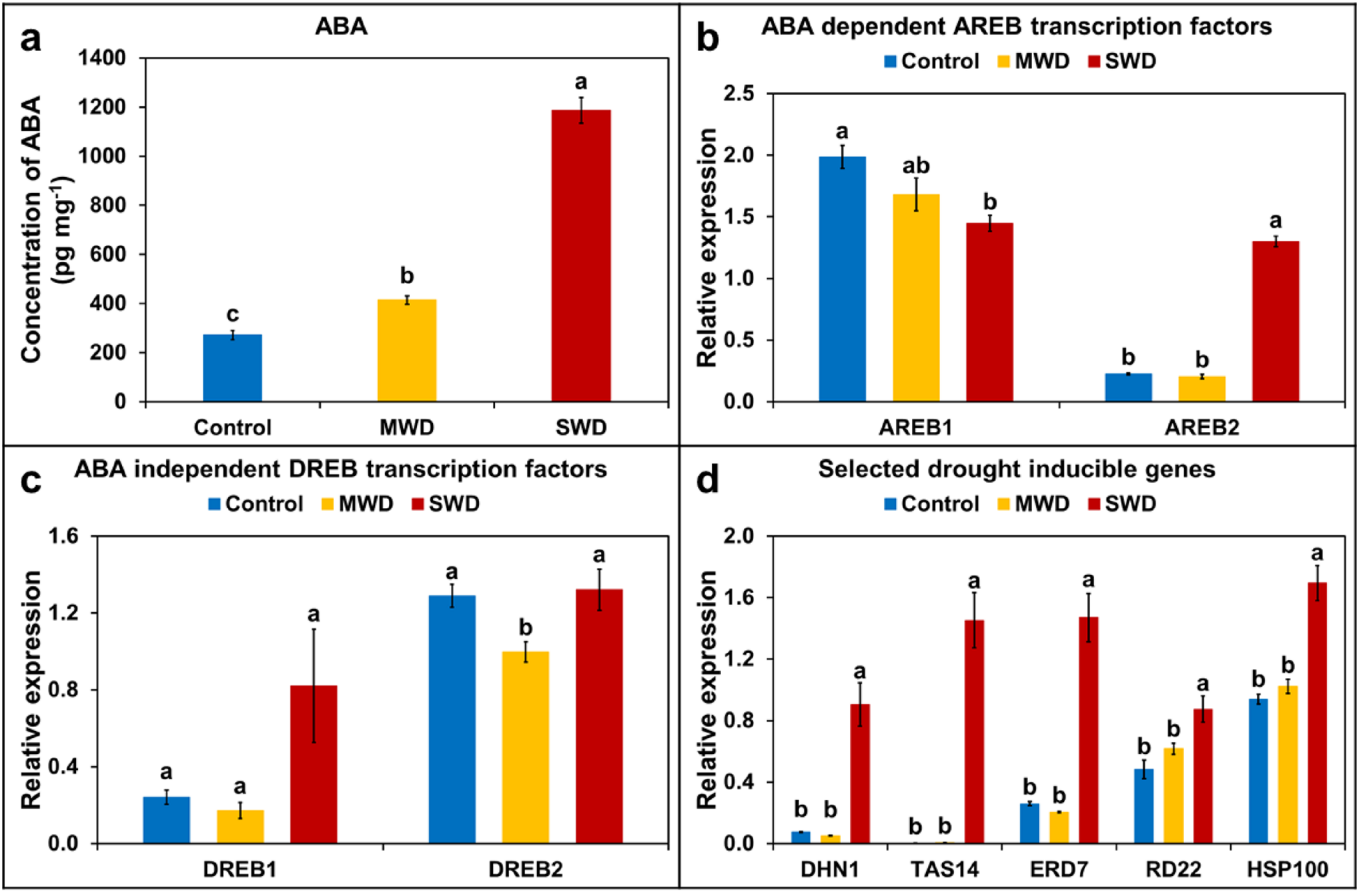
Concentration of phytohormone abscisic acid (ABA) and relative expression of selected drought-responsive genes in potato leaves under Control, Mild (MWD) and Severe (SWD) water deficit conditions. **a** Concentration of ABA in the potato leaves; **b** relative expression levels of ABA-dependent transcription factors in the potato leaves; **c** relative expression levels of ABA-independent transcription factors in the potato leaves and **d** relative expression levels of selected drought-inducible genes in the potato leaves. Bars show means ± SEM of 7 biological replicates. Different letters denote significant differences according to ANOVA followed by a Tukey HSD test (*p* < 0.05; n = 7)

Phytohormone ABA is known to be the key regulator of drought stress responses and, in this regard, leaf vascular tissue appears to be a major contributor to the ABA accumulation which is required for plants to respond more comprehensively to water deficit. In the present study, concentration of ABA significantly increased in potato leaves (Fig. 2a), which corresponds closely with the varying levels of water deficit (MWD and SWD). In comparison to the Control condition, a progressive and significant increase in ABA levels could be observed at MWD, which was even more pronounced at SWD, the latter exhibiting a nearly fourfold increase compared to the Control and a threefold increase compared to MWD (Fig. 2a).

To further understand the effects of variations in water deficit levels on potato plants, selected drought-responsive genes were also examined. These are generally ABA-dependent or ABA-independent, according to whether they rely on ABA for induction. Expression levels of four transcription factors namely *StAREB1*, *StAREB2* (ABA-dependent) and *StDREB1, StDREB2* (ABA-independent) were analysed using real-time PCR (Fig. 2b and c). Of the ABA-dependent transcription factors, *StAREB2* showed a significant upregulation at SWD in comparison to both the Control and MWD, however, no significant induction was observed at MWD when compared to the Control (Fig. 2b). The expression levels of both *StDREB1* and *StDREB2* showed no statistically significant induction under any of the stressed conditions (Fig. 2c). A set of selected drought-inducible genes, such as the LEA and heat-shock protein genes *StDHN1*, *StTAS14*, *StERD7*, *StRD22* and *StHSP100* were further examined. All of these genes showed significant induction in the transcript levels at SWD in comparison to both the Control and MWD (Fig. 2d). However, no significant induction was observed in any of the analyzed genes at MWD in comparison to the Control (Fig. 2d). These results clearly suggest that drought stress perception and signaling varies widely with the degree of applied stress.

### Characterisation of water deficit effects on soil and plant water parameters and tuber production

**Fig. 3:**
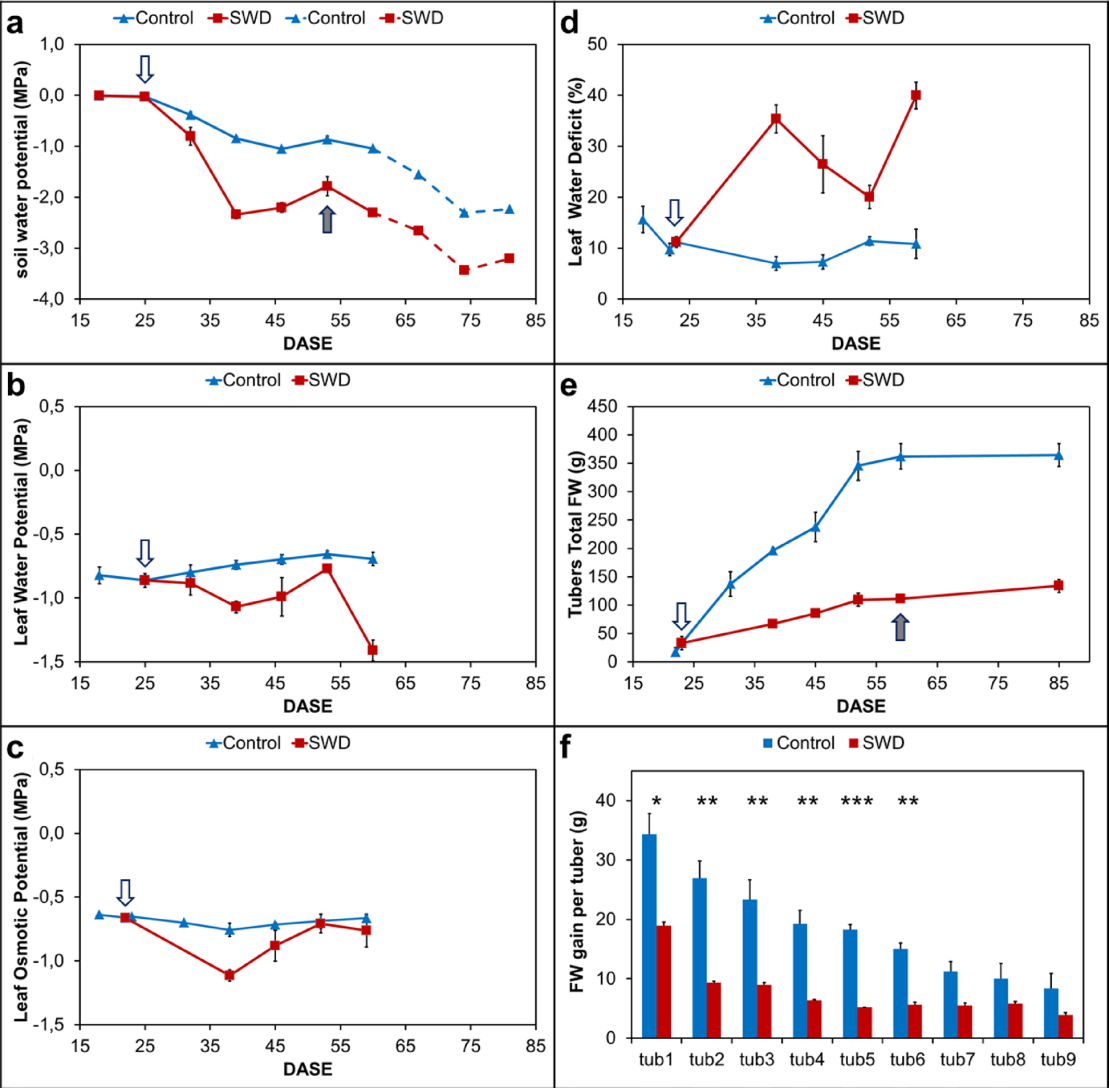
Comparison of the evolution of water parameters and tuber production under Control and Severe Water Deficit (SWD) conditions during filling. **a** Evolution plotted according to Days After Shoot Emergence (DASE) of **a** soil water potential, **b** leaf water potential, **c** leaf osmotic potential, **d** leaf water deficit and **e** total fresh weight of potato tubers from individual plants; **f** fresh weight gain of the nine largest tubers of individual plants, from biggest (tub 1) to smallest (tub 9) during the tuber filling period (38 DASE to harvest). Outline arrow indicates stress application date and full arrow indicates top-kill date. Values are means ± S.E. of 4 independent plants for each condition

In this part, analyses were carried out under Control conditions and under the most severe stress conditions (SWD conditions) that displayed the greatest effects on plant growth and gene expression compared with the Control. Water flux in the soil plant atmosphere continuum is driven by the establishment of a water potential gradient created by leaf transpiration process. Variations in water availability occurring among water stress treatments can be monitored through the characterization of soil water potential. Additionally, the effects of stress on plant water relations can be followed by leaf water potential measurement [26]. As sandy soil was used in this experiment, the main factor influencing soil water potential is soil moisture. Soil water potential was monitored throughout the experiment (Fig. 3a). Under Control conditions, soil water potential was maintained at around 0 MPa from tuber planting up to 25 DASE. It then decreased and remained stable at about − 1 MPa from 39 to 60 DASE. A decrease to − 2 MPa was observed from 60 to 85 DASE corresponding to the withholding of water from top-kill to final harvest. Under SWD conditions, soil water potential decreased when water deficit was induced by water input reduction at 25 DASE. It was maintained at a value close to − 2 MPa (between − 1.78 to − 2.34 MPa) from 39 DASE until top-kill (60 DASE) by the daily adjustment of water management. The more drastic decrease to − 3.43 MPa observed from 60 to 85 DASE corresponded to the withholding of water.

Leaf water potential was simultaneously monitored (Fig. 3b). In Control conditions, unlike soil water potential, leaf water potential remained stable at approximately − 0.75 MPa during the entire measurement period from shoot emergence to top-kill. In SWD conditions, leaf water potential slightly decreased 1 week after water input reduction and reached − 1.1 MPa at 39 DASE. During the period when soil water potential was maintained at around − 2 MPa, a slight increase of leaf water potential was observed close to that seen in the Control plants followed by a decrease to − 1.4 MPa. This corresponded to the increase observed in soil water potential associated with water inputs to maintain soil watering at 20% of field capacity.

The leaf osmotic potential (measured at relative water content) presented a pattern similar to that of leaf water potential (Fig. 3c). In the Control plants, values remained close to − 0.7 MPa for all times of sampling and were not significantly different in SWD plants, except at 38 DASE where leaf water potential showed a slight decrease at − 1.12 MPa. Simultaneously, leaf water deficit remained stable at close to 10% in the Control plants whereas it strongly increased in SWD plants with values ranging between 20 and 40% (Fig. 3d). A comparison of calculated osmotic potential at full turgor between the Control and SWD plants revealed no difference indicating that the potato plants analyzed did not carry out osmotic adjustment.

In addition to soil and plant water parameters, the effects of water input patterns were also evaluated for tuber production (Fig. 3e and f). The sum of all tubers Fresh Weight (FW) from individual plants was computed (Fig. 3e). In addition, the FW gain was evaluated for the nine largest individual tubers of each plant during the tuber filling stage (between 38 to 85 DASE) (Fig. 3f). In the Control plants, tuber total FW steadily increased from 22 to 52 DASE, corresponding to the tuber filling stage, reaching 345 g per plant. After 52 DASE and until harvest, this value remained stable. In SWD plants, tuber FW increase was also observed during the same period (22 to 52 DASE). However, the maximum value reached was 111 g per plant corresponding to about a third of the tuber yield obtained in the Control plants.

When plants produced nine viable tubers (defined as having a tuber diameter greater than 25 mm, 38 DASE in this study), FW of the individual tubers was measured for each plant. Then, FW gain was determined for individual tubers, from the largest (tub1) to the smallest (tub9), between this date (38 DASE) and the harvest date (85 DASE) (Fig. 3f). It appeared that in the Control plants, FW gain was proportional to tuber size from the 1st to the 6^th^ tuber while for the last 3 tubers (tubs 7–9) FW gain remained at the same low values. For tuber FW at 38 DASE, this gain corresponded to a factor of 2.5 (tubs 1–2) to 2 (tubs 3–9). In SWD plants, a lower FW gain was observed than in the Control plants. The FW gain in the largest tuber was about 20 g while no variation was observed in other tubers where the gain was below 10 g.

### Characterization of leaf aging and senescence

**Fig. 4:**
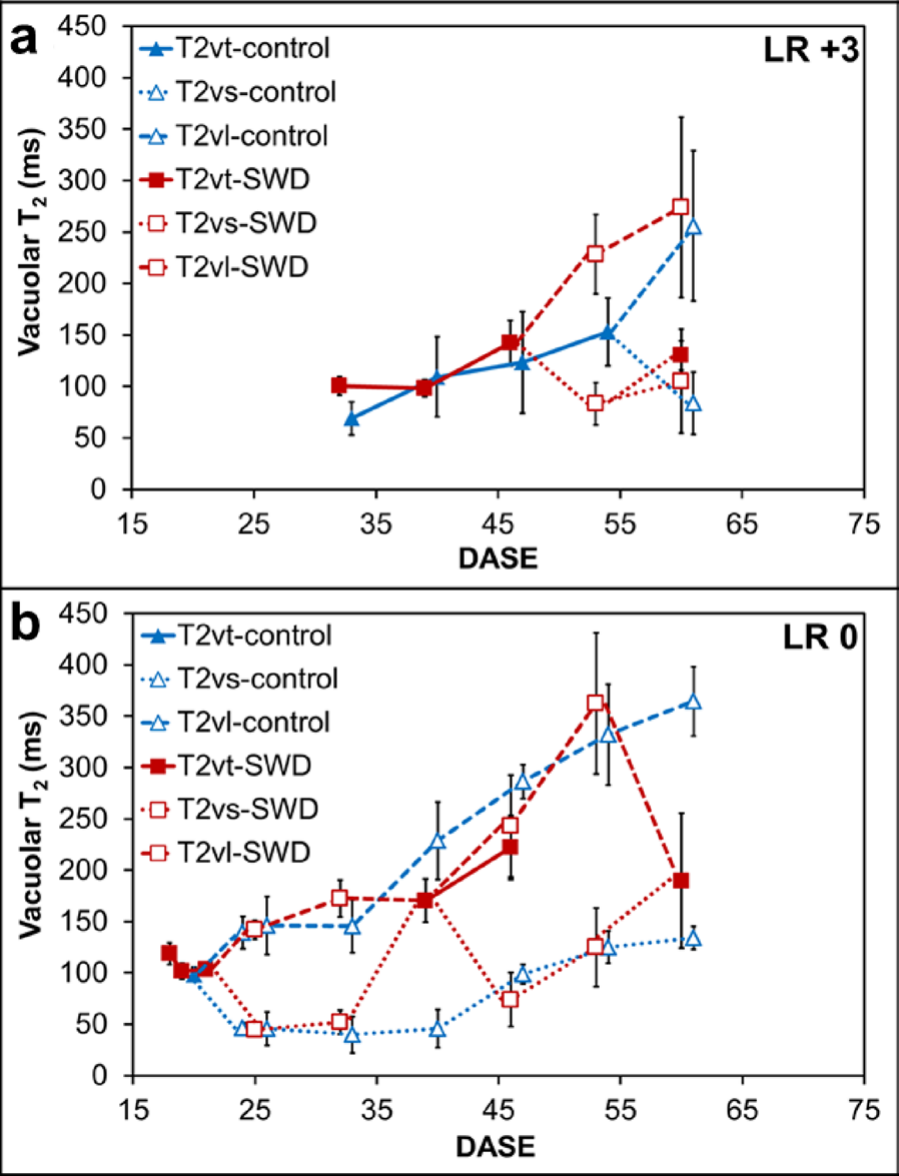
Evolution plotted according to Days After Shoot Emergence (DASE) of NMR relaxation parameter T_2_ of young (**a**) and mature (**b**) potato leaves (referring to the first measurement date) from plants grown under Control and Severe Water Deficit (SWD) conditions. Parameters correspond to vacuolar water from small (vs) and large (vl) mesophyll cells when the NMR signal made it possible to distinguish between them (in well hydrated old leaf tissues). Otherwise, NMR signal corresponds to all (vt) mesophyll cells (in young or dehydrated leaf tissues) (see [13] for further details). Values correspond to averages of maximum 4 leaves. Standard deviation is shown when value corresponds to averages of 3–4 leaves

Changes in T_2_ parameters were monitored for two leaves (Leaf Ranks (LR) 0 and + 3) throughout the six-week plant development period and under Control and SWD conditions. As expected, all T_2_ distributions measured in the leaves displayed several distinct peaks (data not shown). NMR data were interpreted in line with results obtained for oilseed rape, tobacco and escarole [13–15]. In the following, only relaxation peaks associated with the vacuole water pool (either one or two peaks characterized by the longest T_2_ relaxation times, depending on leaf age and condition) are presented. In all Control plants analyzed (Fig. 4a and b), the progression of structural changes associated with leaf development was reflected by the pattern of T_2_ evolution in accordance with previous results obtained from well-watered plants. Indeed, the T_2_ of the single vacuolar peak observed in young leaves (Fig. 4a) increased with leaf development and split, in senescing leaves, into two peaks corresponding to the palisade (large vacuoles) and spongy (small vacuoles) layers (Fig. 4b). The split occurred at 62 DASE for leaves at LR + 3, while it occurred at 24 DASE for LR 0 leaves following the bottom-up progression of structural changes associated with leaf development throughout the canopy. Note that for LR + 3 (Fig. 4a), the pattern of T_2_ evolution was not verified for one of the four leaves analyzed at each developmental stage (data not shown). In SWD plants, the evolution pattern of the vacuolar T_2_ observed in young leaves (LR + 3) was similar to that observed in the Control plants. Differences were observed only for the time when the split occurred, corresponding to 54 and 46 DASE for the Control and SWD plants respectively. In old leaves, the pattern of vacuolar T_2_ evolution was complex. Up to 32 DASE, the T_2_ pattern was similar in the Control and SWD plants. At 39 DASE, the palisade and spongy vacuolar peaks merged, then split again at 46 DASE and finally re-merged at 60 DASE. At 46 DASE, only half of the data made it possible to distinguish between the vacuoles of the two leaf tissues while for the other half, peaks remained fused.

### In-situ monitoring of tuber growth

**Fig. 5:**
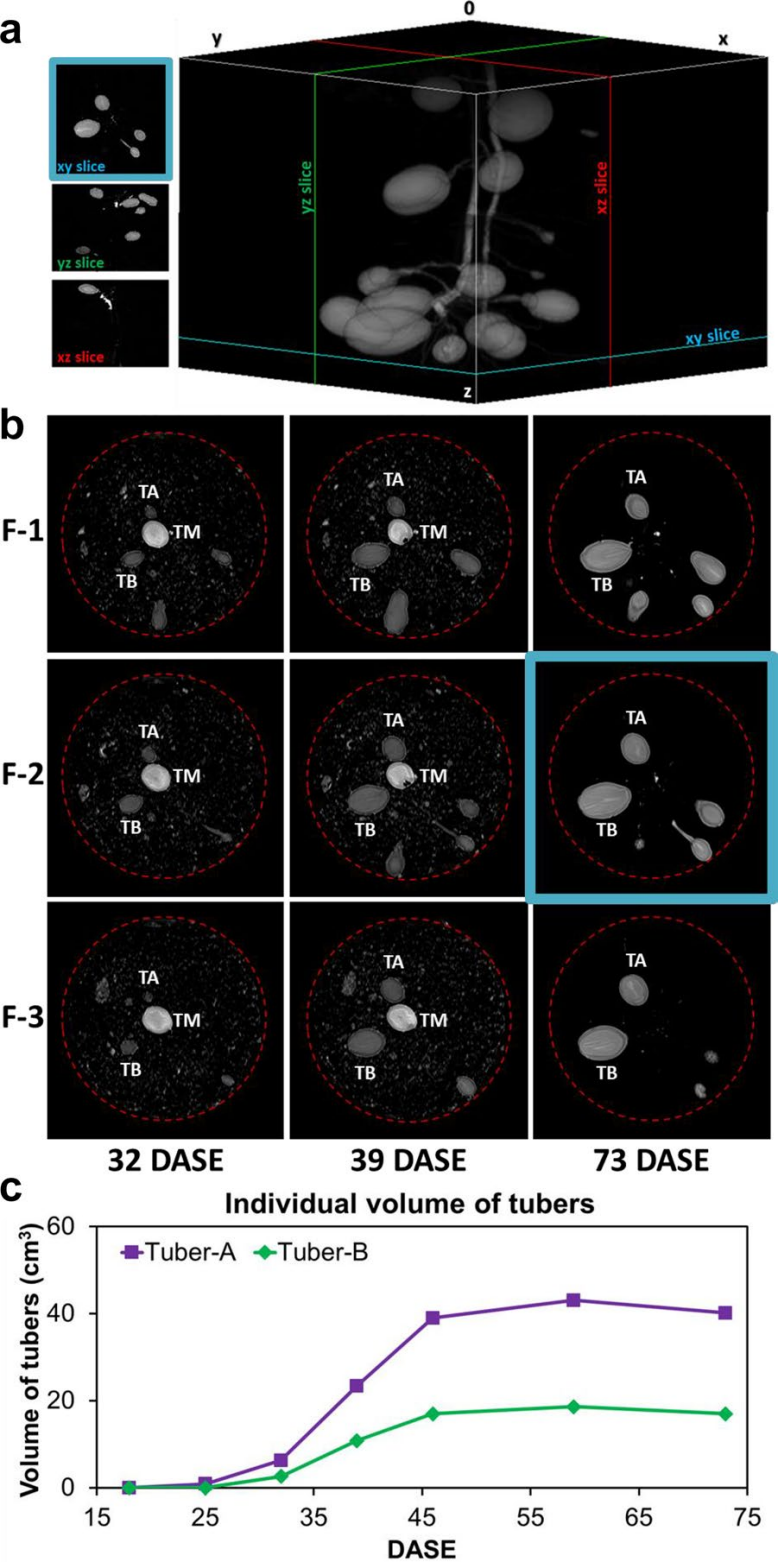
**a** 3D view reconstructed from 3D TSE MRI images of the underground organs of a potato plant 73 Days After Shoot Emergence (DASE). **b** 3D TSE MRI images (TE = 37 ms, TR = 400 ms, BW = 199 Hz/pixel, 1 × 1x1.1 mm^3^ voxel) of potato tubers in soil at three growing stages determined according to days after shoot emergence (columns from left to right: 32, 39, and 73 DASE). Rows correspond to 1 mm frames (from top to bottom: F-1 to F-3 separated by 5 mm). The mother tuber (TM) was placed in the middle of the container, seen in the center of the picture. Dashed red lines represent the edge of the container. Differences in soil aspect are due to different levels of soil hydration on the measurement day. The xy slice in **a** corresponds to F-2 at 73 DASE in **b**. **c** Individual growth curves of Tubers A and B

MRI was used as a non-destructive method to monitor the growth of potato tubers while still in their culture medium. MRI images of the underground organs of the plant in the container enabled access to the number, volume and spatial distribution of tubers during the initiation, filling and maturation phases. Figure 5a provides an example of the 3D MRI of the potato tubers inside the pot reconstructed from the sequential MRI images (see Additional file 2: Video S1). In Fig. 5b, sequential observation of the images along the vertical axis reveals the 3D structure of the tubers, while the horizontal axis corresponds to the growing time (32, 39 and 73 DASE, see also the corresponding video in Additional file 2). The images represented in Fig. 5b were chosen in order to illustrate the individual kinetics of tubers A (TA) and B (TB). From 32 to 39 DASE, tuber volumes increased markedly and reached their final values at 73 DASE (final measurement day before harvest), shown here for TA and TB (Fig. 5c). Indeed, the volume of Tuber A was 6.2 cm^3^ at 32 DASE, increasing to 23.4 cm^3^ at 39 DASE and reaching 40.2 cm^3^ at 73 DASE. The volume of Tuber B was 2.6 cm^3^ at 32 DASE, increasing to 10.8 cm^3^ at 39 DASE and reaching 17.0 cm^3^ at 73 DASE.

The mother tuber (TM, placed in the middle of the container and seen in the center of the picture) shrank over time and was completely depleted at 73 DASE. Based on the visual observation of all images, no direct relation was identified between tuber volumes and position in the pots.

**Fig. 6:**
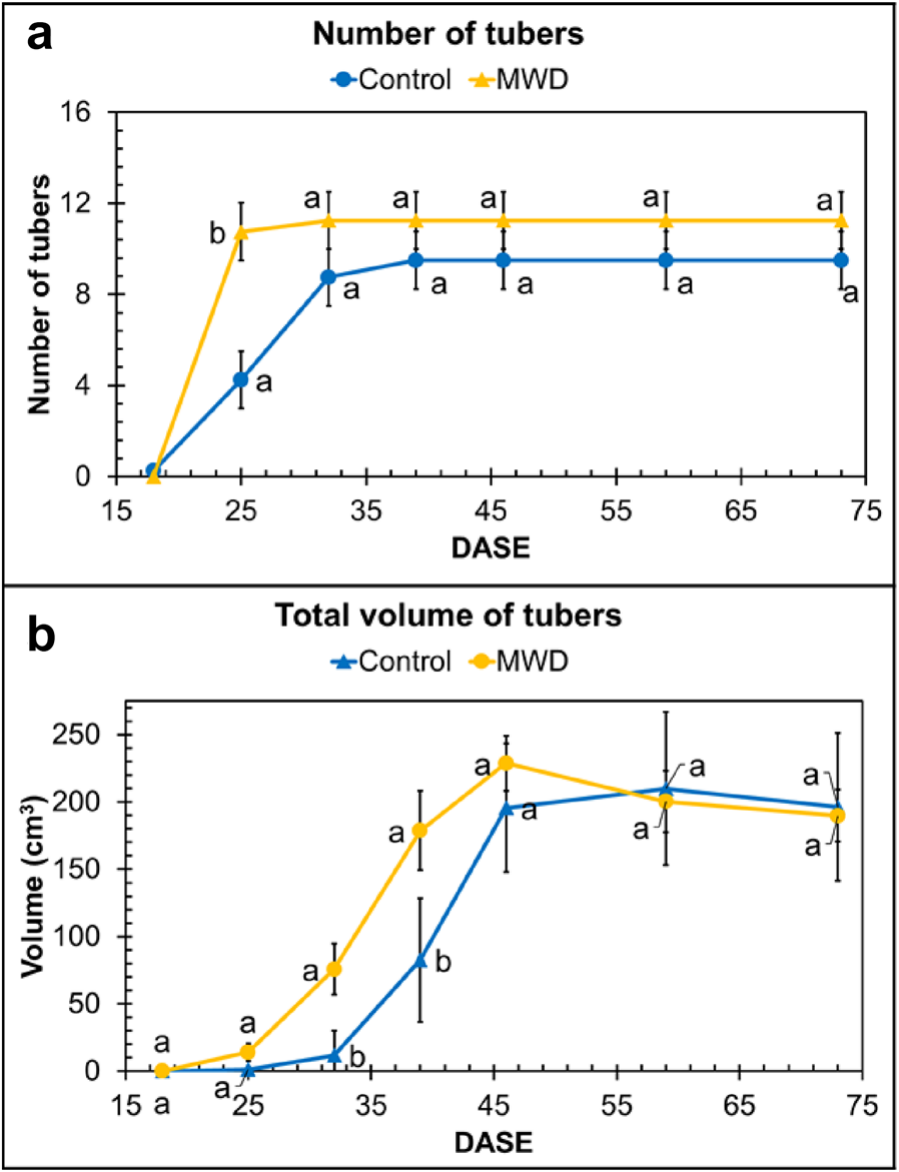
Evolution of mean number of tubers (**a**, **b**) and mean total volume of tubers (**b**) during growth of the Control and MWD potato plants expressed as Days After Shoot Emergence (DASE). For the same DASE, values labelled with different letters denote significant differences according to ANOVA followed by a Tukey HSD test (*p* < 0.05; n = 4)

The MRI images made it possible to detect tubers with diameters greater than 3 mm. However, tubers with a diameter of under 15 mm at 73 DASE were not included in the further analyses, as they are not taken into account for tuber yield. The mean number of tubers for each condition (Control and MWD) was plotted against time (Fig. 6a). At 25 DASE, all tubers from MWD plants were already initiated whereas tubers from Control plants were initiated between 18 and 32 DASE. After 32 DASE, tubers under both conditions had entered the filling phase and no significant difference between conditions was observed for tuber number.

In order to achieve a better comparison between the Control and MWD conditions, the total volume of tubers was calculated for each of 4 plants per condition and the average total volume of tubers was then determined for each condition and plotted in terms of DASE (Fig. 6b). The total volume of tubers was significantly higher (*p* < 0.05) in MWD plants than in the Control plants at 32 and 39 DASE. From 46 DASE, no significant difference (*p* > 0.05) in total tuber volumes of the Control and MWD plants was observed. Figure 5a and b were obtained from the individual growth kinetics of the potato tubers of each plant. Additionally to Tubers A and B (Fig. 5c), individual growth curves for the six biggest tubers of each plant from the Control and MWD plants are shown in Additional file 1: Figure S1.

## Discussion

### Specified irrigation systems combined with high-throughput phenotyping allowed monitoring of each drought treatment

Three specified water deficit levels (MWD, SWD, Control) were applied throughout the entire growth period of potato plants and were evaluated using different methods to explore stressed plant responses. The combination of phenotypical, physiological, metabolomics, molecular and imaging methods enabled the characterization and elucidation of the stress response not only in the commonly studied aboveground leaf systems but also in underground tubers. First, a clear picture of the shifting levels of water deficit stress (MWD and SWD) throughout the growth period of potato plants was established by high-throughput phenotyping parameters, confirming that plants’ response to water stress varies depending on the intensity and duration of the stress imposed [27, 28]. Magnitudes of shoot-growth decrease revealed by the high-throughput technology described in this work fell within the range of those previously reported in other studies [29].

Following on from this, further studies were performed on the effects of varying stress levels on the potato plants’ perception of water deficit signals by estimating the concentration of ABA. This is a highly relevant marker because of ABA’s status as a stress phytohormone and its role in mediating adaptive responses to stress [24, 29]. Under drought stress, increases in ABA levels in particular have been reported in plants, suggesting that ABA regulates and coordinates various signal transduction pathways [30]. In the present study, the significant increase in ABA levels observed in the leaves corresponded closely to the varying stress levels (MWD and SWD). Subsequently, selected ABA-dependent or ABA-independent drought-responsive and drought-inducible genes were also analyzed. They were found to be induced only under severe stress conditions (SWD) and could be attributed to prolonged stress [31].

### NMR relaxation enabled the characterization of the effects of drought on water distribution between leaf tissue types

Following this first set of measurements comparing MWD and SWD, a second trial focused on plant response under SWD, where adjustments in water supply levels appeared to induce the most contrasted changes compared to optimized irrigation. Under Control conditions, NMR measurements were performed to characterize the water distribution in the leaf tissues according to leaf ranks. Under SWD conditions, it was possible to reproduce data previously reported in the literature on the effects of drought on crop yield and plant water relationships. For instance, a decrease in watering corresponded to a tuber yield loss of 60 to 70% compared with irrigated conditions [29]. Likewise, a decrease in soil water potential to below − 2 MPa was associated with a decrease in leaf water potential and an increase in leaf water deficit values in accordance with those observed previously under similar conditions [30]. However, in the present study, it was possible to show variations in leaf water relations across the growth period that were directly associated with slight variations in water input due to the fine tuning of soil water content. The NMR measurements for the Control plants confirmed the findings of previous studies on the evolution of water distribution in leaf tissues in line with the structural changes associated with development [13]. However, the present study provides the first report of the fact that, under SWD conditions, NMR relaxation has the capacity to reveal variations in water distribution in leaf tissues associated with even minor variations in the hydration levels of the soil.

### MRI enabled characterization of the spatial distribution and growth kinetics of potato tubers and evaluation of the effects of soil water dehydration

In addition to the virtues of being non-destructive, MRI provides a way to access the underground parts of plants that are almost impossible to access using traditional methods. In the present study, MRI provided information on the effects of drought on the number of newly-formed potato tubers and made it possible to monitor individual tuber growth within the pot at all times during the growth period, from tuber filling up to harvest. Under MWD conditions, potato tubers reached volumes equal to those of the Control potatoes at the end of the growth stage (Fig. 6b). However, water deficit significantly affected the growth kinetics in early growth stages, demonstrating that the growth kinetics of potato plants are dependent on their water uptake. Increases in tuber volume (Fig. 6a) were similar under both water regimes. However, this increase started earlier under MWD conditions (18 DASE) than under Control conditions (25 DASE). The slight increase in tuber volume (Fig. 6b) observed during the first phase occurred at tuber initiation and the total volume increase was the result of an increase in tuber number. Under Control conditions, the highest increase observed between 32 and 39 DASE, with an increase of 56% of tuber total volume, corresponded to the tuber filling phase. Under MWD, a similar increase associated with the tuber filling period was observed between 25 and 32 DASE and corresponded to a 45% increase of tuber total volume. At crop maturity, the maximum total volume of the Control plants was reached at 59 DASE whereas for MWD plants, it was reached at 46 DASE. Where these conditions are concerned, it appears that MWD induced early initiation followed by early growth in the tubers compared with Control conditions.

## Conclusion

In the present work, the effect of drought on potato production was assessed adopting a multi-scale approach that combined conventional studies on physiological plant response and gene expression analysis with innovative technologies for high-throughput phenotyping at whole-plant (high-throughput phenotyping using RGB cameras) or organ and tissue levels (NMR and MRI analyses). Of the range of abiotic stresses that affect potato crops (see recent review [12]), the present study focused on the effects of drought on yield and biomass. Water deficit has an impact at a number of different stages of potato growth (see review [27]), so the present work has chosen to concentrate on the effects of dehydration occurring mostly during the tuber-filling phase. This study concludes that the close monitoring of the results of applied water stress, through the precise measurement of soil hydration status combined with the use of phenotyping tools allows precise characterization of the effects of treatment. It does so by controlling variations in plant response by adjusting the physico-chemical environmental conditions that directly impact such responses. NMR relaxation offers a promising technological tool in this approach to the understanding of the effects of water stress on water distribution at tissue and cell level in leaf tissues, and hence for the characterization of plant response to drought. In addition, MRI constitutes a non-destructive and powerful method to monitor stress-induced changes underground, in roots and tubers.

A multi-scale approach of this type could be used to undertake studies on the genetic variability of potato plant responses to drought. It could also serve as a means to evaluate new practices such as the use of seaweed extracts to stimulate plant drought resistance [31].

## Materials and methods

### Plant materials

#### Plant growth conditions

Potato (*Solanum tuberosum*) tubers were produced by GERMICOPA (Quimper, France) under irrigated field conditions and stored while dormant at 4 °C until use for experimentation under controlled or semi-controlled conditions. The variety Rosanna was chosen for its relatively low sensitivity to drought. From a batch of calibrated potatoes (28–35 mm), tubers were selected for homogeneous weight (average 23 ± 2 mg fresh weight). Before planting, they were pre-germinated in the dark at 20 °C for two weeks and tubers with 1 to 2 sprouts were retained. The plants were grown in 25 L plastic pots (Airpot®, 27 cm diameter, 50 cm high) filled with a mixture (Falienor® ref. 992016F1) of sandy loam (40% v/v) and peat moss (60% v/v) with added clay (40 kg/m^3^) and NPK (0.7 kg/m^3^ PG-MIX 14-16-18) (soil solution: pH 5.8 ± 0.2 and Ec [1/1,5] 0,7 mS/cm). A single tuber was placed in each pot at 25 cm depth. All pots were filled with the same quantity of soil (17.4 ± 0.1 kg at soil humidity equivalent to 70% of field capacity). At shoot emergence, two to three stems were present per pot. In the controlled environment, in order to optimize plant phenotyping, thinning was carried out to retain only one stem per pot. Plant measurements began at 15 days after shoot emergence (DASE) and continued until top kill day (55 DASE). Plants were grown under either a controlled environment (using the Roullier high-throughput plant phenotyping platform and a greenhouse equipped with high-pressure sodium lights) or a semi-controlled environment (using an INRAE greenhouse equipped with an air-cooling system, X: 48° 5′ 59.99" Y: − 1° 48′ 0"). In the controlled environment, the temperature regime was 22 (light phase)/19 (dark phase) °C and relative humidity was 70% with a light/dark cycle of 16/8 h. In the semi-controlled environment, plants received natural light with a light/dark cycle of 15/9 h at the start of measurements (0 DASE) and at final harvest (85 DASE), the longest light period being recorded at 50 DASE (16/8 h). During the plant growth period (up to 57 DASE), the temperature regime was 24.2 ± 1.1 (light phase)/14.7 ± 0.5 (dark phase) °C with a relative humidity of 53.7 ± 2.4 (light phase)/90.7 ± 0.7 (dark phase) %. Following top kill, the average temperature increased to 27.1 ± 1.2 (light phase) /17.9 ± 0.3 (dark phase) °C while the relative humidity decreased to 49.0 ± 2.9 (light phase)/85.3 ± 1.0 (dark phase) %.

#### Plant watering regimes

All plants were well watered from planting to 25 DASE. From 25 to 55 DASE, the experimental design comprised three different watering regimes—a well-watered (Control) and mild (MWD) and severe (SWD) water deficits—corresponding to soil humidity levels equivalent to 70 (Control), 40 (MWD) and 20 (SWD) % of field capacity. The amount of water supplied corresponded to around 100 (Control), 50 (MDW) and 30 (SWD) % of evaporative demand. The amount of water to be supplied was determined by automatic weighing twice a day under controlled conditions and by daily manual weighing under semi-controlled conditions. From top kill day (57 DASE) the plants were top killed and watering ceased from 57 DASE to final tuber harvest.

#### Plant sampling

High throughput and MRI phenotyping were performed without plant sampling or destruction. All leaves of those plants used for high throughput phenotyping were collected on the last day of measurement (53 DASE) and were frozen in liquid nitrogen for further analysis (phytohormone analysis and gene expression). For the physiological and NMR measurements, different plants were harvested and used on each measurement date. For all plants, at the stress application date (25 DASE), the youngest leaf (of over 2 cm in size) that had emerged from the apical stem was tagged with a plastic wire and was allocated to rank 0 (LR 0). When the leaf in the third rank above LR 0 emerged, it was also tagged with a wire and referred to as leaf rank 3 (LR + 3). The data presented in the paper were obtained from leaves sampled from LR 0 and LR + 3 for the NMR measurements and from LR 0 for the water relation measurements.

### Physiological measurements

Physiological measurements that provide information concerning plant responses to water treatment are presented in Fig. 3. They include measurements of the soil and leaf water relations, such as soil and leaf water potential, leaf osmotic potential, and leaf water deficit along with measurements concerning tuber biomass.

Soil water potential was monitored using MPS-6 dielectric water potential sensors (Decagon Devices Inc.) placed at a depth of 25 cm (at mid-height and half-way between the center and the rim of the pot). Leaf water potential was quantified using a WP4C dewpoint potential meter (Meter Group Inc.) in “precise mode”. From LR 0, two leaflets were sampled and immediately frozen in order to carry out osmotic potential measurements, while two further leaflets were collected to measure the water deficit [28]. Leaf water deficit (LWD) was calculated as LWD = 1-RWC, where RWC is leaf relative water content [28]. Osmotic potential at RWC was measured on sap expressed from the thawed samples using a Roebling freezing point osmometer (Model 13DR, Roebling, Berlin, Germany).

Tuber fresh weight was obtained from all tubers harvested from the plant at the various measurement dates.

### High-throughput plant phenotyping platform

For the entire growing period, plants were monitored using the high-throughput plant phenotyping platform (in a greenhouse equipped with high-pressure sodium lights) where images of each plant were acquired by an imaging unit twice a week. The imaging unit is made up of top and side high definition RGB cameras and a LED light system (5500 K ± 500 K). For all images of each plant, a custom-made segmentation algorithm based on a machine learning technique was used to determine the mask of the plant and delete the background. Depending on the quality of the segmentation, such mathematical morphology algorithms can be used to filter artefacts and clean raw images. Once all the images had been segmented, the morphological and color parameters were computed. The morphological parameters linked to the development of plant architecture are the convex hull areas and projected areas for the top and side views along with the width and height for the side view. For the color parameter, the ExG index was computed (Excess Green Index Eq. 1). This index intensifies the greenness of the plant for higher values.

1 $$ExG=\frac{2*G-(R+B)}{R+G+B}$$

### Determination of phytohormones using UHPLC-MS/MS

The leaves of potato plants for all tested conditions (Control, MWD and SWD) were harvested at 53 DASE and immediately frozen with liquid nitrogen and stored at − 80 °C for further phytohormonal and molecular analyses.

Phytohormone abscisic acid (ABA) was analyzed using a UHPLC-MS/MS system. For the analysis, 10 mg ground leaf samples were extracted using 70% methanol, 29% H_2_O and 1% formic acid containing isotope-labelled internal standards, and centrifuged at 12,600 rpm to collect the supernatant. After evaporation (SPE Dry 96, Biotage, Uppsala, Sweden), the extract was resuspended in a 2% formic acid solution and purified using a 30 mg/mL SPE ABN express plate (Biotage, Uppsala, Sweden). The phytohormones were eluted with methanol, and the samples were evaporated and resuspended in 200 µL of 0.1% formic acid solution before injection into the system. The separation and detection were carried out using a Nexera X2 UHPLC system (Shimadzu, Japan) coupled to a QTrap 6500 + mass spectrometer (Sciex, Concord, ON, Canada) equipped with an IonDrive turbo V electrospray (ESI) source. Phytohormone separation was carried out by injecting 2 µL into a Kinetex Evo C18 core–shell column (100 × 2.1 mm, 2.6 µm, Phenomenex, Torrance, CA, USA) at a flow rate of 0.7 mL min^−1^ and the column oven was kept at 40 °C. The mobile phases, solvents A and B, were composed of Milli-Q water containing 0.1% formic acid (LCMS grade, Merck, Darmstadt, Germany) and acetonitrile containing 0.1% formic acid (LCMS grade, Fisher Optima, UK) respectively. The analysis was carried out in scheduled MRM in negative mode. All quantitative data were processed using MultiQuant V 3.0.2 software (Sciex, Canada).

### Gene expression analysis using real-time PCR

The harvested frozen leaf samples from the control and stressed potato plants were ground to a fine powder under liquid nitrogen. Total RNA was extracted from 100 mg ground samples using a Nucleospin® 8 RNA kit in accordance with the manufacturer’s protocol (Macherey–Nagel, Düren, Germany). The quality and yield of extracted RNA samples were analysed and checked in a 4200 Tapestation (Agilent Technologies, USA) and were then subjected to simultaneous DNase treatment and cDNA synthesis from 1 μg RNA using an iScript™ gDNA clear cDNA synthesis kit (BioRad, CA, USA). Quantitative RT-PCR (qRT-PCR) analysis was performed on a total volume of 10 μl using Universal SYBR Green Supermix (Bio-Rad, CA, USA) in a Real-Time PCR Detection System (Bio-Rad, CA, USA). The qRT-PCR reactions were obtained in technical triplicates using independent cDNA reactions for each biological replicate and 300 nM of gene-specific primer pairs (Additional file 1: Table S2). The thermal cycler protocol included preincubation at 98 °C for 3 min, first followed by 40 cycles of amplification, each consisting of denaturation for 15 s at 98 °C, then by annealing for 30 s at 60 °C and then by elongation at 72 °C for 15 s with a final 5-min extension at 72 °C. Additionally, a melting curve analysis was performed at the end of each assay to confirm the absence of multiple products or primer dimers. The expression of all candidate genes was normalized against four potato reference genes, namely, *StRPL2, StEF1α, StActin* and *StGAPDH*. Specific primers for all candidate genes were designed using Primer3 software and are listed in Additional file 1: Table S1. Primer efficiency for each gene (target genes and reference genes) was calculated by the standard curve method using the appropriate dilution series. All qPCR expression data were acquired and analyzed using CFX Maestro Software Version 1.0 (BIO-RAD, CA, USA).

### NMR relaxometry

For plants grown under semi-controlled environments, the youngest leaf of each plant at 25 DASE was tagged and considered to be leaf rank 0. NMR measurements were performed on the five to six oldest leaves of each plant. To achieve this, one or two leaves older and three leaves younger than the reference senescing leaf (rank 0) from plants grown under optimal and SWD conditions were analyzed.

Transverse relaxation measurements were performed on a 20 MHz spectrometer (Minispec PC-120, Bruker, Karlsruhe, Germany). Analyses were successively performed on six fully expanded leaves (leaf ranks − 2 to + 3 by reference to the tag) of four individual plants (corresponding to four replicates) for each Control and MWD conditions. In some cases where plants were at later stages of development, no leaves at LR -2 were present on the plant and only 5 leaves were analyzed. Four discs 8 mm in diameter were excised from three external leaflets (12 in all) without either cutting off the leaf or uprooting the plant. To obtain homogeneous tissues, the discs were taken from each side of the central vein as close as possible to the midrib and avoiding lateral veins. The discs were then placed in NMR tubes which were covered with a 3-cm-long Teflon cap to avoid water loss during measurement. The temperature of the samples inside the NMR probe was set at 18 °C. Transverse relaxation times were measured using the Carr-Purcell-Meiboom-Gill (CPMG) sequence with a 90°–180° pulse spacing of 0.2 ms and 64 averages. The number of successive echoes recorded was adjusted for each sample according to its T_2_. The recycle delay of the samples was adjusted following measurement of the longitudinal relaxation time (T_1_) with a fast-saturation-recovery sequence. The measurement time for T_2_ (including spectrometer adjustments and the T_1_ measurement) was about 10 min per sample. The CPMG signal was fitted using Scilab software in accordance with the maximum entropy method (MEM) [Mariette F, Data Handl Sci Technol 1996], which provides a continuous distribution of relaxation time components with no assumption concerning their number. In this representation, the peaks of the distribution are centered on the corresponding most probable T_2_ values, while the peak areas correspond to the intensity of the T_2_ components. For each leaf rank, when the number of T_2_ vacuolar components was not identical across all repetitions (one or two, depending the repetition), NMR data were averaged only in homogenous groups of repetitions, with the same number of vacuolar components.

### MRI acquisition and image treatment

Images of the underground part of the potato plant in pots were recorded on a 1.5 T MRI scanner (Magnetom Avanto, Siemens, Erlangen, Germany) equipped with a circular polarized head array coil. The mother tuber was placed about 25 cm deep in pots with a height of 50 cm. Tubers were allowed to grow under a defined water intake regime (Control and MWD). Measurements were carried out 7 times over 8 months corresponding to the period from the initiation of tuberization until tuber harvest. Pots were placed horizontally inside the MRI tunnel and their centers were carefully marked before the first MRI experiment in order to maintain the same position for subsequent experiments during the growing process. All images were acquired using a standard 3D Fast Spin Echo (FSE) sequence with the following parameters: imaging matrix 256 × 256, Field of View (FOV) 256 × 256 mm^2^, slice thickness 1.1 mm, flip angle 170°, repetition time (TR) 400 ms, echo time (TE) 37 ms, echo train length (ETL) 16, bandwidth (BW) 199 Hz/pixel, 1 average and 50% interpolation in the slice direction. Experiments were conducted at room temperature.

Image processing was performed using Seg3D V2.4.0 (SCI University of Utah). Semi-automated tuber segmentation consisted of three main steps: (i) manual contouring of each tuber (seed) on one slice in two of the 192 coronal-acquired slices; (ii) automatic segmentation using “Segmentation Level Set”, a built-in advanced filter, to expand manually-segmented seeds to include any surrounding pixels that are statistically matched with those within the selection; (iii) manual adjustment of automatically drawn masks, if necessary. Then, a 3D Object Counter plugin in ImageJ V1.53b (National Institute of Health) was applied on the generated masks in order to count the 3D objects (corresponding to tubers) in the image stack and to quantify the volume of each tuber. This allowed the number and spatial distribution of tubers to be determined, and their individual volume to be quantified for the analysis period.

## Supplementary Information

**Additional file 1: Figure S1.** Evolution of individual volume of the six biggest tubers of each plant analyzed by MRI during growth expressed as Days After Shoot Emergence (DASE). The volume was determined from 3D MRI images. Tubers were assigned Tuber-01 to 06 (a to f, respectively) according to their volumes in decreasing order at the last measurement day before harvest (73 DASE). **Table S1.** Groups by ANOVA followed by Tukey HSD test aplied on parameters shown in Fig. 1 (R software, alpha = 5%). **Table S2.** List of primers used for qRT-PCR.

**Additional file 2.** Video, 3D MRI of the potato tubers inside the pot reconstructed from the sequential MRI images.

## Data Availability

All data generated or analyzed during this study are included in this published article (and its additional files). The MRI data presented in this study are openly available in Data INRAE (https://data.inrae.fr/) repository at: https://data.inrae.fr/dataset.xhtml?persistentId=doi:10.15454/SFAXAA).
