# Supplementary material for: A global non-invasive methodology for the phenotyping of potato under water deficit conditions using imaging, physiological and molecular tools
Source: Plant Methods. 2021 Jul 23;17:81. doi: 10.1186/s13007-021-00771-0 (PMC8299642; doi:10.1186/s13007-021-00771-0)
Supplement: Supplementary file 1 — Additional file 1: Figure S1. Evolution of individual volume of the six biggest tubers of each plant analyzed by MRI during growth expressed as Days After Shoot Emergence (DASE). The volume was determined from 3D MRI images. Tubers were assigned Tuber-01 to 06 (a to f, respectively) according to their volumes in decreasing order at the last measurement day before harvest (73 DASE). Table S1. Groups by ANOVA followed by Tukey HSD test aplied on parameters shown in Fig. 1 (R software, alpha = 5%). Table S2. List of primers used for qRT-PCR. [file 13007_2021_771_MOESM1_ESM.docx]

**SUPPLEMENTARY DATA**


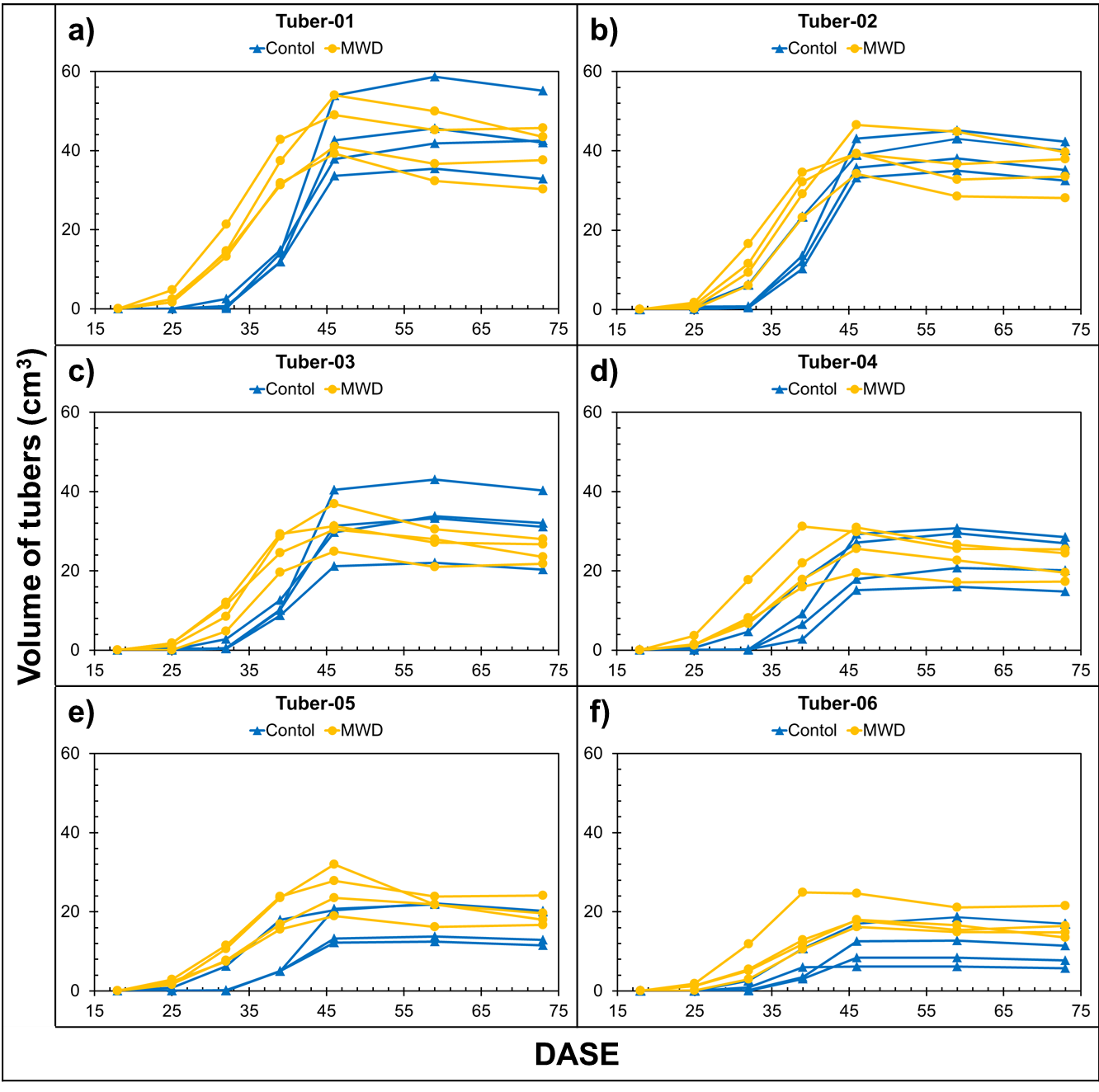


**Supplementary Figure S1**: Evolution of individual volume of the six biggest tubers of each plant analyzed by MRI during growth expressed as Days After Shoot Emergence (DASE). The volume was determined from 3D MRI images. Tubers were assigned Tuber-01 to 06 (a to f, respectively) according to their volumes in decreasing order at the last measurement day before harvest (73 DASE).

**Supplementary Table 1. Groups by ANOVA followed by Tukey HSD test aplied on parameters shown in Figure 1 (R software, alpha = 5%)**

|  | **a) Hull Area**  **Topview** | | | **b) Area**  **Topview** | | | **c) Hull Area**  **Sideview** | | | **d) Area**  **Sideview** | | | **e) Height** | | | **f) Width** | | | **g) ExG** | | |
| --- | --- | --- | --- | --- | --- | --- | --- | --- | --- | --- | --- | --- | --- | --- | --- | --- | --- | --- | --- | --- | --- |
| **DASE** | **Control** | **M**  **W**  **D** | **S**  **W**  **D** | **Control** | **M**  **W**  **D** | **S**  **W**  **D** | **Control** | **M**  **W**  **D** | **S**  **W**  **D** | **Control** | **M**  **W**  **D** | **S**  **W**  **D** | **Control** | **M**  **W**  **D** | **S**  **W**  **D** | **Control** | **M**  **W**  **D** | **S**  **W**  **D** | **Control** | **M**  **W**  **D** | **S**  **W**  **D** |
| **18** | a | a | a | a | a | a | a | a | a | a | a | a | a | a | a | a | a | a | a | a | a |
| **21** | a | a | a | a | a | a | a | a | a | a | a | a | a | a | a | a | a | a | a | b | b |
| **25** | a | a | a | a | a | a | a | a | a | a | ab | b | a | a | a | a | a | a | a | b | b |
| **28** | a | b | b | a | b | b | a | b | b | a | b | c | a | b | c | a | b | b | a | b | b |
| **32** | a | b | c | a | b | c | a | b | c | a | b | c | a | b | c | a | b | c | a | b | b |
| **34** | a | b | c | a | b | c | a | b | c | a | b | c | a | b | c | a | b | c | a | b | c |
| **40** | a | b | c | a | b | c | a | b | c | a | b | c | a | b | c | a | b | c | a | b | c |
| **43** | a | b | c | a | b | c | a | b | c | a | b | c | a | b | c | a | b | c | a | b | c |
| **46** | a | b | c | a | b | c | a | b | c | a | b | c | a | b | c | a | b | c | a | b | c |
| **47** | a | b | c | a | b | c | a | b | c | a | b | c | a | b | c | a | b | c | a | b | c |
| **49** | a | b | c | a | b | c | a | b | c | a | b | c | a | b | c | a | b | c | a | b | c |
| **53** | a | b | c | a | b | c | a | b | c | a | b | c | a | b | c | a | b | c | a | b | c |

**Supplementary Table 2. List of primers used for qRT-PCR.**

| **GENE** | **ACCESSION NO.** | **FORWARD PRIMER** | **REVERSE PRIMER** | **AMPLICON SIZE (BP)** |
| --- | --- | --- | --- | --- |
| ***StAREB1*** | XM_006346349 | 5’-GGCTCAAGGCGGAGTTATG-3’ | 5’-GGGAAGGTGAAAGAGACGATG-3’ | 125 |
| ***StAREB2*** | XM_015312047 | 5’-CAGAACCATCAACCACAGCA-3’ | 5’-ATACCAACCATCCCTACCCTC-3’ | 143 |
| ***StDREB1*** | XM_006358327 | 5’-ACTTGCACGAAACATCAACATC-3’ | 5’-TTATCCCTCCTTCTCACTCCC-3’ | 148 |
| ***StDREB2*** | JN125858 | 5’-AAAGCAGAGGGAACACCAAC-3’ | 5’-GGGAAGAATAAGAACCAAGCCA-3’ | 128 |
| ***StDHN1*** | XM_015304546 | 5’-AGGAGAAATTGCCAGGAGGT-3’ | 5’-GTGCCTTCCATACCATAACCAG-3’ | 85 |
| ***StTAS14*** | XM_015304540 | 5’-TGGCACTCAAGGTAGCGG-3’ | 5’-TCCTCCTCCTGGCATCTTCT-3’ | 175 |
| ***StERD7*** | XM_006359626 | 5’-TGGGGATGTTACTGTGGATAGG-3’ | 5’-GAGACCTTCACTACACCTGAGA-3’ | 180 |
| ***StRD22*** | JX839749 | 5’-CACACAGTTAGCAAGAGCAAAG-3’ | 5’-GGTATCCAAGTGACAAACAGCA-3’ | 93 |
| ***StHSP100*** | XM_006338326 | 5’-GCAAGTTTATGTTGACCAGCC-3’ | 5’-GCCGTGTCTGAAATGCGA-3’ | 105 |
| ***StRPL2*** | DQ252497 | 5’-GAGGGAGAGAGAGAAGAGAGAG-3’ | 5’-GGTGGTGGGTATGGGATTTG-3’ | 100 |
| ***StEF1α*** | AB061263 | 5’-GATGATTCCCACCAAGCCCA-3’ | 5’-TGACAACACCGACAGCAACA-3’ | 107 |
| ***StACTIN*** | DQ252512 | 5’-GTGTGATGGTGGGTATGGGT-3’ | 5’-GGCTTCAGTTAGGAGGACAGG-3’ | 200 |
| ***StGAPDH*** | NM_001288348 | 5’-TGCTCCTATGTTTGTCGTTGG-3’ | 5’-TTCTGAGTGGCTGTGATGGA-3' | 177 |
